# Sleeping together: understanding the association between relationship type, sexual activity, and sleep

**DOI:** 10.5935/1984-0063.20220005

**Published:** 2022

**Authors:** Madeline Sprajcer, Catherine O’Mullan, Amy Reynolds, Jessica L. Paterson, Alysa Bachmann, Michele Lastella

**Affiliations:** 1 Central Queensland University, Appleton Institute - Adelaide - South Australia - Australia.; 2 Central Queensland University, School of Health, Medical and Applied Sciences - Bundaberg - Queensland - Australia.; 3 Flinders University, Flinders Health and Medical Research Institute (Sleep Health) - Bedford Park - South Australia - Australia.

**Keywords:** Sexual Behavior, Family Relations, Orgasm, Sleep

## Abstract

**Objective:**

Insufficient sleep, and particularly difficulties initiating sleep, are prevalent in the community. Treatment for poor sleep typically consists of pharmacological intervention, or cognitive behavioural therapies - which can be both costly and time-consuming. Evidence suggests that sexual activities may positively impact sleep. However, little is known about relationship types, sexual activities, and perceived sleep outcomes. The aim of this study was to explore the association between relationship type (e.g., having a regular, occasional, or casual partner), sexual activity and satisfaction, and perceived sleep outcomes, to identify potential strategies to improve sleep.

**Methods:**

Seven-hundred and seventy-eight participants aged 18 years and over (442 females, 336 males; mean age 34.5 ± 11.4 years) responded to a cross-sectional online anonymous survey at their convenience. Participants were asked about their sleep, sexual activity and satisfaction, and relationship type.

**Results:**

Results from multiple regression analyses with age and gender covariates revealed that shorter sleep latencies were associated with regular relationships (p = 0.030), greater emotional satisfaction with sexual activity (p = 0.029), and increased frequency of orgasm (p < 0.001). Men reported a greater frequency of orgasm than women (p < 0.001).

**Discussion:**

Findings indicate that relationship type may be associated with improved sleep outcomes, including sleep latency. Relationship type should therefore be taken into consideration by clinicians when developing treatment plans for individuals with poor sleep.

## INTRODUCTION

In most western countries, a significant proportion of adults suffer from sleep disturbances. To be specific, over 35% of adults report sleeping poorly on a regular basis. Two of the common complaints of poor sleep relate to the initiation and/or maintenance of sleep. Sleep problems have a significant economic impact due to the cost of medical care, lost work, reduced productivity, and accidents/injuries ^1^. For example, insomnia is estimated to cost the United States up to $150 billion annually, directly and indirectly ^3^. Further, the personal and public health impact of poor sleep is significant. Poor sleep is associated with higher rates of physical health concerns (e.g. cardiovascular disease, metabolic or gastrointestinal disorders)^4,5^, mental health problems ^6^, and negative performance outcomes (e.g. poorer productivity, increased accident and injury rates) ^7^.

Treatment for difficulty initiating and/or maintaining sleep typically involves the use of pharmacological intervention or cognitive therapies ^8^. However, pharmacological interventions are likely to result in tolerance or dependence (and associated withdrawal symptoms upon cessation), and in some cases significant side effects including parasomnias or interactions with other pharmaceuticals ^9^. While Cognitive Behavioural Therapy (CBT) is currently the treatment of choice for many specialists, this treatment tends to be expensive and time consuming (i.e. based on frequent sessions with a trained specialist), which impacts uptake and efficacy ^10^. Therefore, alternative non-pharmaceutical treatment options that are time- and cost-effective may be an attractive prospect.

There is a relationship between sexual activity and sleep ^12,13,14^. From a public health perspective, evidence indicating that sexual activity improves sleep may provide additional support for individuals experiencing sleep problems, without the negative side effects or time/cost associated with traditional treatment. Recent findings suggest that sexual activity, both with and without a partner, may result in positive sleep outcomes – including shorter sleep latency and improved sleep quality ^13^. This largely self-reported finding is supported by animal models, where copulatory activity in male rats is associated with increased slow wave sleep ^15^. There is also a relationship between poorer sleep quality and sexual arousal, which may have a hormonal basis ^14^. Furthermore, sexual activities such as intercourse and masturbation release multiple hormones that promote sleep. Primarily, oxytocin and prolactin have been identified as key hormonal responses to sexual activity. Sexual arousal has also been found to reduce the cortisol (i.e., stress) response. These three hormones have also been independently associated with sleep:

- Increased cortisol is associated with poor sleep quality (e.g. overnight awakenings) via activation of the hypothalamic–pituitary–adrenal (HPA)-axis ^16^.
- Prolactin is thought to be positively associated with sleep, based on inferences made from overnight blood plasma levels ^17^.
- Oxytocin is associated with positive sleep outcomes, including reduced sleep latency and increased sleep efficiency ^18^.

Sexual activity is a fundamental part of most intimate relationships, and couples engage in sexual activity for many reasons – including emotional satisfaction, physical pleasure, stress reduction, reproduction, and expression of emotion ^19^. As factors relating to intimate relationships are likely to be associated with sexual activity (e.g. satisfaction experienced, frequency of sex, emotional intimacy) ^20^, relationship type (e.g. having a regular, occasional, or casual partner) may also impact sleep. This is supported by the impact of family relationship quality on sleep – with more supportive relationships associated with better sleep ^21^. Additionally, there is evidence suggesting that the status of romantic relationships may impact sleep, with partners who have had negative behavioural exchanges with their partners having poorer sleep ^22^. Intimate relationships may take many forms – marriage, cohabitation, dating, and a variety of casual and non-standard relationship models. There is some evidence that relationship type may impact sleep – potentially as a result of emotional ties and attachment or feelings of safety ^23^.

While few studies have included the categorization of relationship type, findings have indicated that couples’ sleep/wake behaviours are likely to be interdependent, such that their bed and wake times are influenced by the physical presence of their partner ^24^. Some studies have indicated that sleeping with a partner may be beneficial for sleep, resulting in increased total sleep time, subjective sleep quality, and sleep efficiency ^25^. This may be due to evolutionary adaptive function that increases physical and emotional security when sleeping in pairs, and thus may reduce arousal levels and increase sleep quality and quantity ^23^. This suggests that there are differences in sleep quality and duration based on how comfortable members of a couple are with each other (i.e. the degree of physical and emotional security that is experienced), which may be associated with the type of relationship they are in.

Given that intimate relationships can strongly predict physiological and psychological wellbeing ^26^, it is surprising that no study has examined the influence of relationship type on sexual activity, relationship and sexual satisfaction, and sleep. This is particularly important to understand given the high proportion of adults who currently report difficulty falling asleep or insufficient sleep. The aim of this study was therefore to explore the association between relationship type, sexual activity and satisfaction, and self-reported sleep outcomes.

## MATERIAL AND METHODS

Seven-hundred and seventy-eight participants aged 18 years and over (442 females, 336 males; mean age 34.5 ± 11.4 years) volunteered to complete an online anonymous survey at their convenience between October 2016 and June 2017. Participants were recruited through social media platforms (i.e. Twitter, Facebook) and professional networks of Australian researchers, though participants were not required to be Australian residents. A link to the online survey was posted and participants were encouraged to repost as a form of snowballing sampling. Ethical approval was obtained through the University Human Research Ethics Committee (H16/09-260). Informed consent was obtained from all individual participants included in the study.

### Survey

The survey instrument included pre-validated items derived from the Australian Study of Health and Relationships ^27^ and the Pittsburgh Sleep Quality Index ^28^. The survey contained questions relating to sleep, wake and sexual behaviours and demographic information such as age, gender, sexual orientation, and relationship type. Demographic information collected included age (years), gender, current sexual relationship, and number of children living in the home. Sexual relationship type bins can be seen in Table 1. Participant s were also asked to provide a percentage response to the question, “How honest were you in your answers to the questionnaire?” (0 = not at all honest, 100 = extremely honest).

**Table 1.** Relationship type, sexual activity, and satisfaction response bins.

Question	Original response bins	Adjusted response bins
Current sexual relationship (this might be different to your marital status)	Regular partner Regular partner but not live-in Occasional partner Casual/One Night Stand No partner	Regular partner Regular partner but not live-in Occasional or casual partner No partner
Over the past month, how frequently have you had sex?	More than once a day Daily 5-6 times a week 3-4 times a week 2-3 times a week Once a week Once every 2 weeks Once every 3 weeks Once Never	Daily or more than daily 4-6 times a week 1-3 times a week Once a month to once every 2 weeks Never
Over the past month, how frequently do/did you orgasm from sex with a partner	Never Seldom Half of the time Most of the time Every time	Never or seldom Half of the time Most of the time Every time
Over the past month, how emotionally satisfying has sex been for you?	Extremely satisfying Very satisfying Moderately satisfying Slightly satisfying Not at all satisfying	Extremely or very satisfying Moderately satisfying Slightly satisfying Not at all satisfying

#### Sexual activity and satisfaction.

The survey adapted questions from the Australian Study of Health and Relationships relating to participant’s sexual activity and satisfaction ^27^. Questions focused on sexual activity and satisfaction over the previous month. Responses were binned to ensure statistical assumptions were not violated within analyses. Questions and response bins can be seen in Table 1.

Participants were informed that sex was defined as sexual intercourse (vaginal or anal), oral sex, or manual stimulation of the genitals by a partner. These sexual activity and satisfaction variables were chosen as they cover the physiological aspects of sex hypothesised to impact sleep (i.e. sexual activity and orgasm frequency), along with the psychological aspects (i.e. emotional satisfaction).

#### Sleep

Participants were asked questions about their typical sleep. A truncated version of the Pittsburgh Sleep Quality Index – a widely used measure of sleep quality – was used to understand participant sleep outcomes ^29^. Questions were phrased asking about sleep timing and duration “over the past month”. Participants were asked to reflect on the past month and respond to questions regarding bedtime (AM/PM), time taken to fall asleep, and hours of actual sleep. Participants were also asked to rate how satisfied they were with their sleep (both quality and amount) over the previous month (1 = extremely satisfied to 5 = not at all satisfied).

### Statistics

Data were analysed using R (version R-4.0.0) ^30^. Pearson *X*^2^ analyses were conducted to determine relationships between relationship type and sexual activity and satisfaction outcomes (i.e. emotional satisfaction, sexual activity frequency, and orgasm frequency). Multiple regression analyses were performed to investigate the associations between relationship type, sexual activity and satisfaction, and sleep outcomes (total sleep time and sleep latency). Predictor variables examined in independent models included relationship type, degree of post-sexual activity emotional satisfaction, orgasm frequency, and sexual activity frequency. Simultaneous multiple regressions were performed using dummy coded variables for variables that were not continuous (e.g. relationship type). Sleep variables (total sleep time and sleep latency) were included as outcome measures. All regression analyses included gender and age (bins: 18 – 25, 26 – 35, 36 – 45, 46 – 55, 56 or older) as covariates. Statistical significance is denoted at the .05 level. Due to the sensitive subject matter, no question fields were mandatory. As such, some proportions in the below tables do not tally to 100%. Listwise exclusion was done for models where data were missing, with a maximum removal rate of 9% of respondents.

## RESULTS

### Demographics

There were 778 responses to the online survey. Demographics can be seen in Table 2. Of the participants who completed the survey, 683 (87.8%) provided information about sexual orientation. As heterosexuality was reported by 92.1% of these participants (n = 629) analyses do not include sexual orientation. The mean honesty score was 98.0 (± 5.9) out of a possible 100.

**Table 2.** Participant demographics.

Demographic information	Total n	%
Age		
18-24	151	19.4%
25-34	301	38.7%
35-44	144	18.5%
45-54	107	13.8%
55+	53	6.8%
Gender		
Male	328	42.2%
Female	430	55.3%
Relationship type		
Regular partner (live in)	474	60.9%
Regular partner (not live in)	102	13.1%
Occasional or casual partner	59	7.5%
No partner	47	6.0%
Sexual Orientation		
Heterosexual	629	80.8%
Homosexual	12	1.5%
Bisexual	41	5.3%
Other	1	0.1%

### Relationship type and sexual activity

Pearson’s chi-square analyses were performed to determine the association between different relationship types and how emotionally satisfying sex was over the previous month, sexual activity frequency, and orgasm frequency. These analyses indicated that there were significant differences in outcomes based on relationship type. Having a regular partner was associated with having higher rates of emotional satisfaction and frequency of orgasm. Sample size was too small to examine the differences by partner with frequency of sexual activity (see Table 3).

**Table 3.** Relationship type and sexual activity and satisfaction outcomes over the previous month*.

	Regular partner	Regular partner (not live in)	Occasional or casual partner	No partner	Chi-square analysis
Total number	474	102	59	47	
Emotional satisfaction					Χ^2^(12) = 169.90, p < 0.001
Extremely satisfying	129 (27.2%)	32 (31.4%)	6 (10.2%)	0 (0.0%)	
Very satisfying	184 (38.8%)	45 (44.1%)	18 (30.5%)	3 (6.4%)	
Moderately satisfying	97 (20.5%)	15 (14.7%)	17 (28.8%)	4 (8.5%)	
Slightly satisfying	37 (7.8%)	3 (2.9%)	11 (18.6%)	4 (8.5%)	
Not at all satisfying	23 (4.9%)	5 (4.9%)	6 (10.2%)	20 (42.6%)	
Frequency of sexual activity					Χ^2^(12) =202.48, p < 0.001
Daily or more than once a day	20 (4.2%)	3 (2.9%)	2 (3.4%)	0 (0.0%)	
3 – 6 times a week	87 (18.4%)	31 (2.9%)	5 (8.5%)	1 (2.1%)	
1 – 3 times a week	215 (45.4%)	49 (48.0%)	19 (32.2%)	3 (6.4%)	
Once/month - once/2 weeks	131 (27.6%)	17 (16.7%)	32 (54.2%)	5 (10.6%)	
Never	20 (4.21%)	2 (2.0%)	1 (23.7%)	38 (80.9%)	
Frequency of orgasm					Χ^2^(9) = 94.83, p < 0.001
Never or seldom	69 (14.6%)	15 (14.7%)	16 (27.1%)	23 (48.9%)	
Half of the time	50 (10.5%)	9 (8.8%)	7 (11.9%)	2 (4.3%)	
Most of the time	120 (25.3%)	45 (44.1%)	18 (30.5%)	3 (6.4%)	
Every time	229 (48.3%)	32 (31.4%)	17 (28.8%)	2 (4.3%)	

*Note that reported sexual activity did not include masturbation. In cases of low cell frequency rates, the likelihood ratio correction was reported (DescTools package for R). Bracketed percentages denote proportion of participants reporting each relationship type. Percentages may not add to one hundred due to non-response of some participants to certain questions.

### Orgasm frequency and gender

**Figure 1.**
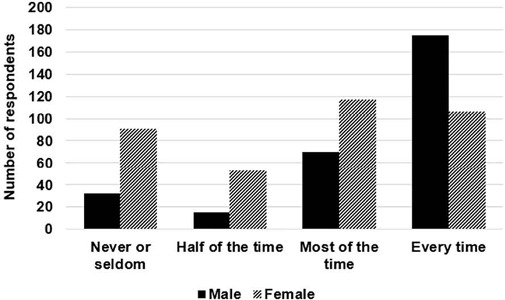
Reported orgasm frequency (over the past month) and gender.

There was a statistically significant difference in reported orgasm frequency between men and women, χ^2^ ^3^ = 70.67, p < 0.001. Differences can be seen in Figure 1.

### Relationship type and sleep

#### Sleep latency.

**Figure 2.**
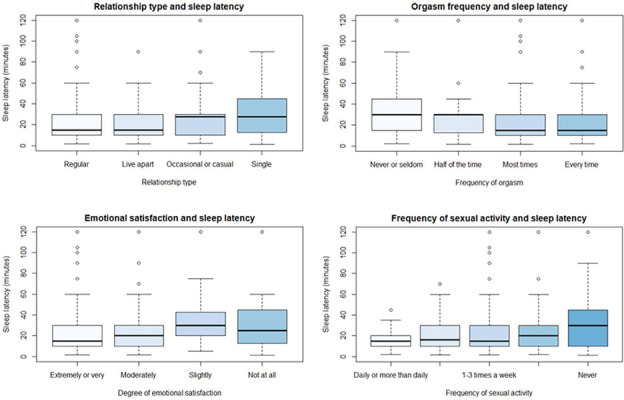
Association between relationship type, emotional satisfaction, orgasm frequency, and frequency of sexual activity and sleep latency.

A simultaneous multiple regression analysis with dummy coded relationship type variables (4) was performed to determine the association between relationship type and sleep latency. Age (binned as described above) and gender were included as covariates. The association between relationship type and sleep latency was significant (F_(8, 665)_ = 2.15, p = 0.030), however only 1.4% of the variance in sleep latency was explained (R^2^ = 0.014). This analysis indicated that individuals with occasional or casual partners take 10.5 (SE = 3.1) minutes longer to fall asleep than individuals who live with a regular partner. See Table 4 and Figure 2.

**Table 4.** Predictive value of relationship type and sexual activity sleep outcomes adjusted for gender and age.

Predictors		β	SE	95% CI	
				Lower	Upper	p
Sleep latency (minutes)						
Relationship type	Regular partner**			Reference group		
	Regular partner (not live in)	-2.65	3.14	-8.82	3.53	0.400
	Occasional or casual partner	10.53	3.96	2.76	18.30	0.008*
	No partner	8.12	4.34	-0.41	16.65	0.062*
Emotional satisfaction	Extremely or very satisfying**			Reference group			
	Moderately satisfying	3.82	2.76	-1.61	9.24	0.168
	Slightly satisfying	10.22	3.97	2.43	18.01	0.010*
	Not at all satisfying	8.73	4.06	0.75	16.70	0.032*
Orgasm frequency	Every time	-12.03	3.11	-18.15	-5.92	<0.001*
	Most of the time	-9.33	3.22	-15.66	-3.00	0.004*
	Half of the time	1.03	4.16	-7.14	9.20	0.804
	Never or seldom**			Reference group		
Frequency of sexual activity	Daily or more than daily**			Reference group		
	3 – 6 times a week	5.04	6.20	-7.12	17.21	0.416
	1 – 3 times a week	8.78	5.89	-2.79	20.35	0.137
	Once/month – once/2 weeks	11.03	6.03	-0.813	22.87	0.068
	Never	17.18	6.72	3.98	30.38	0.011*
Total sleep time (hours) Relationship type	Regular partner**	Reference group				
	Regular partner (not live in)	-0.36	0.52	-1.38	0.66	0.487
	Occasional or casual partner	-0.49	0.66	-1.78	0.80	0.458
	No partner	-0.45	0.71	-1.84	0.95	0.529
Emotional satisfaction	Extremely or very satisfying**	Reference group				
	Moderately satisfying	-0.47	0.47	-1.39	0.46	0.324
	Slightly satisfying	-0.90	0.67	-2.22	0.42	0.181
	Not at all satisfying	-0.74	0.68	-2.08	0.59	0.272
Orgasm frequency	Never or seldom**	Reference group				
	Half of the time	-1.04	0.72	-2.45	0.37	0.146
	Most of the time	-0.67	0.55	-1.76	0.41	0.221
	Every time	-0.50	0.53	-1.55	0.54	0.345
Frequency of sexual activity	Daily or more than daily	Reference group				
	3 – 6 times a week	0.34	1.04	-1.71	2.39	0.742
	1 – 3 times a week	0.51	0.99	-1.44	2.46	0.609
	Once/month – once/2 weeks	-0.02	1.01	-2.01	1.97	0.983
	Never	-0.22	1.12	-2.42	1.98	0.842

* Denotes statistical significance

** β statistics are presented in comparison with level 1 coded variables.

When assessed by gender, it was found that there was no significant relationship between sleep latency and relationship type for men (F_(7, 283)_ = 1.13, p = 0.345). For women, the relationship was significant (F_(7, 375)_ = 2.60, p = 0.012).

#### Total sleep time.

**Figure 3.**
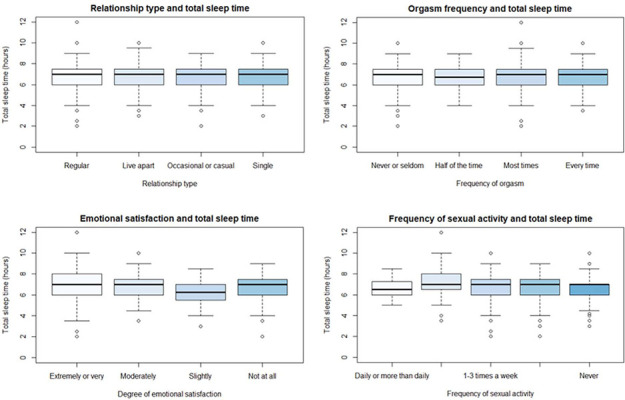
Association between relationship type, emotional satisfaction, orgasm frequency and frequency of sexual activity and total sleep time.

Relationship type was not associated with self-reported total sleep time (F(8, 655) = 0.51, p = 0.853, Figure 3).

### Emotional satisfaction and sleep

#### Sleep latency.

There was a significant relationship between emotional satisfaction and sleep latency (F(8, 643) = 2.16, p = 0.029), which explained 1.4% of the variance in sleep latency (adjusted R2 = 0.014). See Figure 2.

Compared with individuals who reported that they were extremely or very emotionally satisfied, those who reported being slightly satisfied took on average 12.0 (SE = 4.3) minutes longer to fall asleep (p = 0.005). Similarly, those who reported no emotional satisfaction at all took 10.5 (SE = 4.4) minutes longer to fall asleep than individuals who reported that they were extremely or very emotionally satisfied. See Table 4.

However, when each gender was examined individually, no significant impact of emotional satisfaction was seen (males - F_(7, 278)_ = 1.20, p = 0.304; females - F_(7, 358)_ = 1.68, p = 0.114.

#### Total sleep time.

There was no significant relationship between emotional satisfaction and total sleep time, with gender and age as covariates (F(8, 633) =0.75, p < 0.652). See Figure 3.

### Orgasm frequency and sleep

#### Sleep latency.

There was a significant relationship between orgasm frequency and sleep latency (F_(8, 641)_ = 3.56, p < 0.001) that explained 3.1% of the variance in sleep latency (adjusted R^2^ = 0.031, Figure 2). Individuals who reported having an orgasm “every time” fell asleep 12.0 (SE = 3.1) minutes faster than those who reported never or seldom having an orgasm (p = 0.004). Similarly, those who reported having an orgasm “most of the time” fell asleep on average 9.3 (SE = 3.2) minutes faster than those who reported never or seldom having an orgasm (p < 0.001). See Table 4.

When assessed by gender, it was found that there was a significant relationship between sleep latency and orgasm frequency for both men (F_(7, 278)_ = 2.42, p = 0.020) and women (F_(7, 356)_ = 2.68, p = 0.010).

#### Total sleep time.

There was no significant relationship between orgasm frequency and total sleep time, F_(8, 631)_ = 0.67, p = 0.722, see Figure 3.

### Sexual activity frequency and sleep

There was no significant relationship between frequency of sexual activity and sleep latency (F_(9, 665)_ = 1.79, p = 0.066), or total sleep time, (F_(9, 655)_ = 0.58, p = 0.811). See Figure 2 and 3.

## DISCUSSION

The aim of this study was to explore the potential for sexual activity to improve sleep, by examining the associations between relationship type, sexual activity and satisfaction, and perceived sleep outcomes. Participants in regular relationships (regardless of living situation) reported falling asleep faster, higher post-sex emotional satisfaction, and more frequent orgasms. Furthermore, there were associations between orgasm frequency, emotional satisfaction, and sleep latency, suggesting that some aspects of self-reported sexual activity are associated with perceived positive sleep outcomes. Initial analyses examined how relationship type impacts sexual activity. Findings indicated that individuals in regular (either live in or live apart) relationships reported higher emotional satisfaction with sexual activity than those who were single, or in occasional or casual relationships. Similarly, individuals in regular relationships were more likely to report a high frequency of orgasm (73.6% - 75.5% of participants in regular relationships reported having an orgasm ‘most of the time’ or ‘every time’), compared to 59.3% of individuals in occasional or casual relationships, or 10.7% of individuals who were not in a relationship. Gender was also associated with orgasm frequency, with men reporting significantly higher rates of orgasm than women.

Perceived sleep latency and total sleep time were assessed in the context of relationship type. Individuals who cohabit with regular partners reported falling asleep 10.5 minutes faster than those with occasional or casual partners. However, it must be noted that for men, there was no significant difference in sleep latency based on relationship type – this difference is driven by female participants. A sleep latency decrease by 10.5 minutes (as seen in the present study) is significantly greater than the differences seen in Multiple Sleep Latency Tests when individuals with insomnia are compared with healthy individuals (4-8 minutes), which may suggest clinical implications ^31^. There was also a non-significant trend for individuals with regular partners who live separately to report falling asleep faster than those who were in occasional or casual relationships, or those who were single. Sleep latency was also impacted by the degree of emotional satisfaction reported in relation to sexual activity over the previous month. These data suggest that being in a regular relationship is associated with greater emotional satisfaction and also independently with the ability to fall asleep faster. It may be that regular relationships are associated with greater comfort and partner responsiveness (i.e. feeling understood, cared for, and validated), which leads to more positive sleep outcomes ^32^. Further, shorter sleep latency may result from reduced physiological arousal, as sleeping next to a regular partner can improve physical and emotional security, causing a down regulation of arousal levels and an increase in sleep quality and duration ^24^. From an evolutionary perspective, the desire to fall asleep after sexual activity is associated with emotional bonding and expressions of affection, which may also explain the observed relationship between shorter sleep latencies and regular relationships ^33^.

The notion that regular/cohabitational relationships are associated with longer or more efficient sleep indicates that not being in a relationship, or being in an unstable relationship (i.e. a casual or occasional relationship), may negatively impact sleep ^34^. Being in a relationship that is not regular may result in increased arousal and/or rumination – both of which are known to result in poorer sleep ^35,36^. Increased arousal may be caused by either positive or negative affect (or both) associated with new relationships (e.g. excitement, nervousness, insecurity, etc.), or relationship breakups (e.g. anxiety, stress, rumination). Therefore, understanding relationship type and associated emotional state may be useful in identifying underlying causes of poor sleep ^9^. This information may assist clinicians in determining treatment strategies based on personal circumstances. For example, clinicians may be able to identify those individuals at higher risk of poor sleep based on relationship type and reported sexual outcomes, and therefore provide tailored sleep management strategies. Appropriate sleep management strategies should be the focus of future research.

Individuals who reported having an orgasm ‘most of the time’ or ‘every time’ reported falling asleep significantly faster than other participants (9.3 ± 3.2 and 12.0 ± 3.1 minutes faster, respectively). This may relate to the release of multiple sleep facilitating hormones post-orgasm ^37,38,39^. Specifically, the release of oxytocin post-orgasm reduces cortisol and is associated with improved sleep ^16,18^. This is supported by previous research that found an association between sleep quality and sexual satisfaction ^14^. This suggests that orgasm may be an effective strategy to help individuals who report difficulty falling asleep. As such, pre-sleep sexual activity (or masturbation) may be a cost-effective, non-pharmaceutical strategy for managing poor sleep, regardless of relationship type.

There are several key limitations of the present study. As this study was cross-sectional in nature, we are unable to make inferences about the causal relationships between sleep outcomes and sexual activity. Additionally, the completion rate among respondents was 66% indicating that a non-response bias may be of concern. Future research using a randomised, controlled trial approach will help to determine these direct relationships. Furthermore, while surveys can be used to determine perceived sleep outcomes, they do not provide objective measures of sleep. Future research should include physiological measures of sleep to obtain objective, and ecologically valid data (e.g. via at-home polysomnography or actigraphy). In addition, while the present study identified that relationship type, emotional satisfaction, and sexual activity are associated with perceived sleep outcomes, further research is necessary the interplay between relationship dynamics, contributors to emotional satisfaction and poor sleep. The present study also indicated that a higher frequency of self-reported orgasm was associated with more positive sleep outcomes. However, we do not know the timing of sexual activities relative to sleep opportunity, and are therefore limited in our ability to draw conclusions about the direct impact of orgasm frequency (and associated hormonal changes) on sleep outcomes. It would also be useful for future studies to address relationship quality and duration to identify additional factors linking relationship type and sleep outcomes ^40^.

It is important to note that while statistical significance was seen in many analyses, the variance explained by each factor (e.g. relationship type, emotional satisfaction) was reasonably small. This is likely due to the multi-factorial nature of human relationships, physiology, and psychology. This study is one of the first to investigate the relationship between sexual activity and sleep, and as such was limited in capacity to include the variety of personal factors that may impact sleep. For example, this study did not capture genders outside of the male/female binary, or individuals who have more than one partner. Given the complex nature of human sexuality, gender, and other factors, future research is necessary to determine how these factors interact, and may impact sleep.

Sleep outcomes (sleep latency in particular) were associated with relationship and sexual factors within the present study. These data show that regular relationships (i.e. long-term relationships with or without cohabitation) were associated with increased post-sexual activity emotional satisfaction, increased frequency of orgasm, and shorter sleep latency. Therefore, relationship type should be considered by clinicians when implementing tailored intervention strategies for individuals with difficulty initiating sleep. Furthermore, both emotionally satisfying sexual activity and orgasm frequency were positively associated with sleep outcomes. As such, engaging in satisfying sexual activity may be associated with perceived benefit for individuals who struggle to initiate sleep. However, additional research is required regarding the direct physiological relationships between orgasm frequency and sleep outcomes, in addition to objectively measured sleep within the context of relationship type and quality.
